# Reference values for T, B and NK human lymphocyte subpopulations in adults

**DOI:** 10.1016/j.dib.2017.04.019

**Published:** 2017-04-21

**Authors:** P.A. Apoil, B. Puissant-Lubrano, N. Congy-Jolivet, M. Peres, J. Tkaczuk, F. Roubinet, A. Blancher

**Affiliations:** a Laboratoire d׳Immunogénétique Moléculaire, EA 3034, Université Paul Sabatier, Toulouse III, France; b Laboratoire d’Immunologie, CHU de Toulouse, France; c EFS Pyrénées-Méditerranée, Toulouse, France

**Keywords:** Lymphocyte subpopulations, Immunophenotyping, Reference ranges, Age, Sex

## Abstract

The data presented in this paper are reference ranges for frequencies of thirty-eight subpopulations of T, B and NK lymphocytes, established from a cohort of 253 healthy blood donors aged from 19 to 67. When relevant, the influence of age or sex was taken into account to calculate these reference values. This article is related to the research article entitled “Influence of age, sex and HCMV-serostatus on blood lymphocyte subpopulations in healthy adults” (Apoil et al., 2017) [1]. Immunophenotyping data obtained from each individual is made publicly available for extended analyses.

**Specifications Table**

**Table:** 

**Subject area**	Biology
**More specific subject area**	Human Immunology
**Type of data**	Tables
**How data was acquired**	Immunophenotyping by multicolour flow cytometry
**Data format**	Reference values for human lymphocytes are presented as percentages (for subpopulations) or absolute counts (all T lymphocytes, T CD4+, T CD8+, B and NK cells); counts and subpopulation frequencies for each blood donor are communicated as a transparency document.
**Experimental factors**	Peripheral whole blood anticoagulated with EDTA
**Experimental features**	Samples were labeled with 4 distinct antibody panels: four 8-colour panels were used to study subpopulation frequencies and a 4-colour panel was dedicated to absolute counts; reference values were calculated in accordance with the Clinical and Laboratory Standards Institute (CLSI) recommendations. Non-parametric Mann-Whitney test was used to evaluate the impact of age and sex on these subpopulations.
**Data source location**	Toulouse, Midi-Pyrénées, France
**Data accessibility**	The data are available in this article

**Value of the data**

• Reference values of 38 distinct subpopulations of T, B and NK lymphocytes were established through the study of a large population sample of healthy adults, in accordance with CLSI standards.
• These reference values are adequate for interpreting clinical laboratory results from young adults and middle-aged patients.
• Low- frequency subpopulations (T cells expressing intermediate levels of CD4 or CD8 molecules or positive for NK-related markers) were included in this study.
• Reference ranges were adjusted for age or sex when these parameters impact the values.

## 1 Data

Data in the following Tables present the reference values for 38 distinct human T, B or NK lymphocyte subpopulations. When sex or age has a significant impact on these subpopulations, separate reference values are given for male, female, younger (19–44) or older (45–67) individuals. Data are expressed either as absolute numbers of cells in G/L (a), or as the percentage of cells relative to total CD3+ (b), CD3+ CD4+ (c), CD3+ CD8+ (d) T lymphocytes, Tregs (e), total B (f) or NK cells (g); DN: CD4-CD8- double-negative T cells; DP: CD4+CD8+ double-positive T cells.

**Table:** 

**Sub-population** *(T lymphocytes)*	**All** mean (ref. values)	**Males** mean (ref. values)	**Females** mean (ref. values)	**Age≤44** mean (ref. values)	**Age>44** mean (ref. values)
CD3+ ^(a)^	1473 (700–2508)	1387 (675–2491)	1560 (787–2533)	1545 (783–2532)	1412 (699–2213)
CD3+ CD4+ CD8− ^(a)^	928 (464–1721)	841 (394–1620)	1017 (573–1815)	*no influence of age*	
CD3+ CD4− CD8+^(a)^	405 (135–852)	*no influence of sex*		441 (157–881)	370 (131–825)
CD4+/CD8+ *ratio*	2.6 (1–6.2)	2.4 (1–6)	2.7 (1–6.7)	2.3 (0.9–4.2)	2.9 (1–6.7)
CD3+ CD4+ CD8+ DP ^(b)^	0.4% (0.09–1.65)	0.3% (0.07–1.1)	0.5% (0.1–2)	*no influence of age*	
CD3+ CD4− CD8− DN ^(b)^	7% (1.7–21.4)	8.3% (1.8–27.8)	5.6% (1.5–14.9)	8.2% (2.2–25)	5.8% (1.4–20.3)
CD3+ CD4− CD8^low (b)^	2.9% (1.05–5.9)	3.3% (1.41–6.6)	2.6% (1–4.7)	*no influence of age*	
CD3+ CD4+ CD8^low (b)^	0.3% (0.05–1.6)	*no influence of sex*		0.3% (0.05–1.6)	0.3% (0.05–1.6)
CD4+ Naïve ^(c)^ (62L+ 45RA+ 27+ 28+)	43.1% (17.8–66.3)	*no influence of sex*		46.3% (19.9–67.8)	39.8% (14.4–65.2)
CD4+ Central memory ^(c)^ (62L+ 45RA− 27+ 28+)	32.8% (19.4–51.9)	*no influence of sex*		33.9% (7.6–62.5)	31.3% (15.3–52.8)
CD4+ Effector memory ^(c)^ (62L− 45RA+/− 27+/− 28+)	16.7% (7.4–31.9)	17.8% (8–33.7)	15.6% (6.7–27.7)	16% (6.7–28.5)	17.5% (8–35.3)
CD4+ EMRA ^(c)^ (62L− 45RA+ 27−28−)	1.6% (<0.01–14)	*no influence of sex*		0.1% (0–2)	3.4% (0–22.4)
CD8+ Naïve ^(d)^ (62L+ 45RA+ 27+ 28+)	36% (7.5–66.8)	*no influence of sex*		40.6% (6–73.4)	30.2% (1.5–65.5)
CD8+ Central memory ^(d)^ (62L+ 45RA− 27+ 28+)	9.6% (3.4–22.4)	*no influence of sex*		8.1% (3.4–16.8)	11% (3.5–28.6)
CD8+ Effector memory 27+ (62L− 45RA− 27+ 28+/−) ^(d)^	18.9% (6–38.9)	*no influence of sex*		17.3% (5.4–34.4)	20.5% (6–43.4)
CD8+ Effector memory 27- (62L− 45RA− 27− 28+/−) ^(d)^	4.7% (0.4–19)	*no influence of sex*		3.2% (0–23.9)	6.6% (0.7–72.6)
CD8+ EMRA pE1+pE2 (62L− 45RA+ 27+28+/−) ^(d)^	8.6% (2.5–21.2)	9.3% (2.1–22.2)	7.8% (2.5–18.3)	7.6% (2.4–16.5)	9.5% (2.5–22.4)
CD8+ EMRA ^(d)^ (62L− 45RA+ 27− 28−)	9.9% (0.3–32.2)	*no influence of sex*		4.7% (0.1–37.5)	16.5% (1.6–52.5)
Tregs ^(c)^ (4+ CCR4+ 45RA− 127− 25++)	2.9% (1.3–5.5)	*no influence of sex*		2.7% (1.4–5.1)	3.2% (1.0–5.8)
HLA-DR+ Tregs ^(e)^ (Tregs HLA-DR+)	26% (10.3–43.1)	*no influence of sex*		24.4% (9.7–38.3)	28% (10.1–49)
HLA-DR+ CD4+ memory^(c)^ (4+ 45RA− HLA-DR+)	3.2% (0.9–7.7)	*no influence of sex*		2.9% (0.9–6.3)	3.5% (0.8–8.2)
HLA-DR+ CD8+ memory^(d)^ (8+ 45RA− HLA-DR+)	10.2% (2.9–25.4)	*no influence of sex*		*no influence of age*	
CD3+ NKB1− NKp30+ ^(b)^	0.5% (0–4.4)	0.4% (0–4.3)	0.5% (0–5.1)	0.4% (0–2.2)	0.5% (0–3.4)
CD3+ 56+ ^(b)^	5.5% (1.1–14.9)	*no influence of sex*		*no influence of age*	
CD3+ 16+ ^(b)^	2.4% (0.3–8.1)	*no influence of sex*		2.8% (0.3–9.9)	2% (0.3–7)

**Sub-population** *(CD45+, B & NK cells)*	**All** mean (ref. values)	**Males** Mean (ref. values)	**Females** mean (ref. values)	**Age≤44** mean (ref. values	**Age>44** mean (ref. values)
Lymphocytes CD45+ ^(a)^	2012 (959–3644))	1904 (959–3644)	2120 (1290–3485)	*no influence of age*	
CD19+ B lymphocytes ^(a)^	247 (92–515)	233 (92–437)	260 (91–536)	*no influence of age*	
CD19+ naïve ^(f)^ (IgD+ CD27−)	65% (44–84)	66.6% (44–84)	63.5% (44–84)	*no influence of age*	
CD19+ Transitional ^(f)^ (CD24+ CD38+)	6.2% (1.7–13.8)	6.6% (0.2–12.9)	5.8% (1.5–13.6)	*no influence of age*	
CD19+ Plasmablasts ^(f)^ (CD38+++ CD24−)	1.3% (0.2–5)	*no influence of sex*		1.7% (0.2–7.4)	1.0% (0.1–3.3)
CD19+ Memory ^(f)^ (IgD− CD27+)	10.9% (1.9–13.4)	9.8% (1.1–21.8)	12% (3.7–26.3)	*no influence of age*	
CD19+ Switched ^(f)^ (IgM− IgD− CD27+/−)	16.4% (4.8–33.2)	15.2% (4.9–31.7)	17.6% (5–35.2)	*no influence of age*	
CD19+ CD5+ ^(f)^	9.5% (2.4–20.8)	*no influence of sex*		*no influence of age*	
CD19+ Marginal zone ^(f)^ (IgM high IgD^low^ CD27+)	14.6% (4.8–32)	*no influence of sex*		*no influence of age*	
NK cells ^(a)^ (CD45+ CD16+CD56+)	253 (82–594)	282 (87–633)	246 (70–557)	*no influence of age*	
NK cells CD16^low^CD56^high (g)^	6.4% (1.1–17.7)	*no influence of sex*		7.4% (1.1–19.2)	5.3% (1.2–14.8)
*ratio NK CD16*^*low*^*/CD16*^*high*^	0.07 (0.01–0.2)	*no influence of sex*		0.08 (0.01–0.2)	0.06 (0.01–0.2)
NK cells HLA-DR+ ^(g)^	4.1% (1.1–13.8)	*no influence of sex*		*no influence of age*	
NK cells NKp30+ ^(g)^	74% (17.1–95.6)	*no influence of sex*		*no influence of age*	
NK cells NKp46+ ^(g)^	9.7% (1.9–27.3)	*no influence of sex*		10.7% (2.8–27.4)	8.7% (1.3–23.5)

## 2 Experimental design, materials and methods

### 2.1 Cohort assembly

Healthy blood donors aged 19–67 (median age 44 y.o.) were recruited between 2011 and 2013 in Toulouse (*Etablissement Français du Sang Pyrénées-Méditerranée*, Southwest of France). All subjects were negative in serological tests for blood-transmissible infections (HIV, hepatitis B and C, HTLV, syphilis) and were exempt from any pathology or treatment which could interfere with leukocyte parameters (history of cancer or of autoimmune disease, active or recent systemic infection, immunosuppressive or immunomodulating therapy, severe allergy, or a vaccine administered less than 3 months ago). For each enrolled blood donor, a single 7 mL EDTA tube of peripheral whole blood was collected between 8 a.m. and 11 a.m. Reference values were calculated from a cohort of 253 individuals, adjusted for *sex ratio* and frequency of HCMV-seropositivity, which was made from a larger cohort of 283 blood donors (complete immunophenotyping data for these 283 individuals is presented in Appendix A: Supplementary material). Details about the composition of this cohort are available online in Supplementary material from [1].

### 2.2 Immunophenotyping and cytometry data analysis

Immunophenotyping was performed by multicolour flow cytometry: samples were labeled with 4 distinct antibody panels and absolute counts of T, B and NK cells were determined by using BD Trucount® tubes. Cytometric data was acquired on a BD CANTO II® flow cytometer (BD Biosciences, Le Pont De Claix, France) and was analyzed with BD Diva and FlowJo® software (LLC, Ashland, OR). Antibody panels and gating strategies are detailed in Ref. [1]. Mean values and reference ranges were calculated according to CLSI guidelines [2] (including suspected outliers in the calculation) by using the Reference Value Advisor software [3], from the entire population sample, or from subgroups of more than 120 individuals (males/females and younger/older). To evaluate the influence of age and sex, Mann–Whitney non-parametric tests were carried-out after removal of outliers, and the corrected ranges are indicated when *p*<0.05.

## Funding sources

This work was supported by Paul Sabatier University (Toulouse, France), Région Midi-Pyrénées and by the French Ministry of Higher Education and Research.

## Appendix A Supplementary material

Supplementary material: This table presents individual cytometric data of lymphocyte subpopulations (relative percentages or absolute counts) acquired from 283 healthy blood donors from the Southwest of France. Age, sex and CMV serostatus are indicated. Detailed information about antibody panels and gating strategies can be found in Ref. [1].
